# Personality Development in Emerging Adulthood—How the Perception of Life Events and Mindset Affect Personality Trait Change

**DOI:** 10.3389/fpsyg.2021.671421

**Published:** 2021-06-10

**Authors:** Jantje Hinrika De Vries, Maik Spengler, Andreas Frintrup, Patrick Mussel

**Affiliations:** ^1^Personality Psychology and Psychological Assessment, Freie Universität Berlin, Berlin, Germany; ^2^Division HR Diagnostics AG, Stuttgart, Germany

**Keywords:** personality development, life events, big five, mindset, emerging adulthood

## Abstract

Personality changes throughout the life course and change is often caused by environmental influences, such as critical life events. In the present study, we investigate personality trait development in emerging adulthood as a result of experiencing two major life events: graduating from school and moving away from home. Thereby, we examined the occurrence of the two life events *per se* and the subjective perception of the critical life event in terms of valence. In addition, we postulate a moderation effect of the construct of mindset, which emphasizes that beliefs over the malleability of global attributes can be seen as predictors of resilience to challenges. This suggests that mindset acts as a buffer for these two distinct events. In a large longitudinal sample of 1,243 people entering adulthood, we applied latent structural equation modeling to assess mean-level changes in the Big Five, the influence of life events *per se*, the subjective perception of life events, and a moderating role of mindset. In line with maturity processes, results showed significant mean-level changes in all Big Five traits. While no changes in the Big Five dimensions were noted when the mere occurrence of an event is assessed, results indicated a greater increase in extraversion and diminished increase in emotional stability when we accounted for the individual's (positive/negative) perception of the critical life event. In case of extraversion, this also holds true for the moderator mindset. Our findings contribute valuable insights into the relevance of subjective appraisals to life events and the importance of underlying processes to these events.

## Introduction

People change as they age. Individuals experience not only physical but also psychological changes across the entire lifespan. However, the exact course of internal and external changes depends on various criteria. In recent years, researchers have expended considerable effort in studying how personality develops across the lifespan; this has, in turn, incited a controversy about the stability and variability of specific personality traits. Personality traits are considered to be relatively stable individual differences in affect, behavior, and/or cognition (Johnson, 1997). Whereas, the Big Five traits of conscientiousness and agreeableness appear to be rather stable and continuously increase across adulthood, levels of openness to experience appear to change in an inverted U-shape function, which increases between the ages of 18 and 22 and decreases between 60 and 70 (McCrae and Costa, 1999; Roberts and DelVecchio, 2000; Specht et al., 2011). Furthermore, some studies have shown that trait change can be associated with particular life stages. For example, the findings of Roberts and Mroczek (2008) suggest that young adults tend to exhibit increases in traits that are indicative of greater social maturity. More specifically, in emerging adulthood, the average individual experiences an increase in emotional stability, conscientiousness, and agreeableness (Arnett, 2000; Roberts et al., 2006; Bleidorn, 2015), and self-esteem (Orth et al., 2018), while openness to experience seems to decrease in advancing age (Roberts et al., 2006). Taken together, this comprises evidence that personality develops throughout the lifespan and consequently, several theories have been introduced to explain when and why personality change occurs (e.g., Cattell, 1971; Baltes, 1987; Caspi and Moffitt, 1993; McCrae and Costa, 1999; Roberts and Mroczek, 2008).

### Critical Life Events

Theory and research support the idea that personality can change as a result of intrinsic factors such as genetics and extrinsic factors such as the environment around us (Bleidorn and Schwaba, 2017; Wagner et al., 2020). More specifically, there is ample evidence that personality is linked to certain external influences such as critical life events (e.g., Lüdtke et al., 2011; Bleidorn et al., 2018). These can be defined as “transitions that mark the beginning or the end of a specific status” (Luhmann et al., 2012; p. 594) and include leaving the parental home or major changes in one's status such as employment or duty. These transitions often require adaptation processes involving new behavioral, cognitive, or emotional responses (Hopson and Adams, 1976; Luhmann et al., 2012, 2014). Profound adaptations are assumed to have lasting effects, as “life events can modify, interrupt or redirect life trajectories by altering individuals' feelings, thoughts and behaviors” (Bleidorn et al., 2018, p. 83). Building upon this assumption, many studies have sought to determine how certain Big Five traits change because of critical life events. For instance, increases in emotional stability were found to result from transitioning into one's first romantic relationship (Lehnart et al., 2010). Emotional stability might also increase in anticipation of gain-based events such as childbirth or paid employment, which, in turn, lead to increases in conscientiousness and openness to experience (Denissen et al., 2018).

In the present study, we focus on two critical life events that are highly relevant for emerging adults: moving away from home and graduating from school. Both events represent a personal development milestone for the transition into adulthood and are typically associated with great educational or occupational challenges (Arnett, 2000; Pusch et al., 2018). Few studies have highlighted these two events and how they influence life trajectories in emerging adulthood. Lüdtke et al. (2011) focused on the broader superordinate section of work-related life events and personality change and found that the transition from high school to college, university, or vocational training is associated with substantial normative increases in emotional stability, agreeableness, and conscientiousness. With regard to graduation from school, Bleidorn (2012) found significant mean-level changes in certain Big Five traits over an observation period of 1 year. Specifically, senior students experienced increases in conscientiousness, agreeableness, and openness after graduation. In a later review by Bleidorn et al. (2018), the authors found that graduation constitutes an almost universal life event in Western societies and that related change in adult personality is likely to be observable, because young adulthood is a period in which personality traits have been shown to be most open to change (Roberts and DelVecchio, 2000; Lucas and Donnellan, 2011).

There are fewer investigations into the personality effects of moving away from home. Pusch et al. (2018) compared age differences in emerging vs. young adults and found that, among other life events, leaving the parental home did not reveal significant age effects with respect to personality change. However, they found significant age-invariant effects for individuals who left their parental home recently, indicating positive changes in agreeableness. Jonkmann et al. (2014) investigated living arrangements after college with regard to personality differences and found that, for example, the choice of living arrangement (living with roommates vs. living alone) predicted the development of conscientiousness and—to a lesser extent—openness and agreeableness. Similarly, according to a study by Niehoff et al. (2017), living and studying abroad after college led to increases in extraversion, agreeableness, and emotional stability. Interestingly, Specht et al. (2011) found a significant sex effect on leaving the parental home and argued that only women become more emotionally stable when moving. Taken together, this evidence suggests that moving away from home is a major life event that has not yet been deeply investigated but represents a distinct developmental task that has the potential to shape individuals' personalities.

### The Perception of Life Events

While these studies provide valuable information about the impact of critical life events, one important issue has been hitherto neglected. Many past studies have focused on life events *per se*, but comparatively little effort has been made to examine the subjective appraisal of such events and its effect on the processes underlying personality change (Roberts, 2009). Moreover, methodological approaches to life events are sometimes misleading, because the valence of experienced events is rated by either researchers or other people who cannot sufficiently reflect inter- and intra-individual experiences of events (Headey and Wearing, 1989; Kendler et al., 2003; Luhmann et al., 2020). However, there is ample evidence that people perceive the same event or situation very differently. For example, according to a comprehensive review of person-situation transactions by Rauthmann et al. (2015), situations can be characterized by their physical (e.g., location, activity, persons) and/or psychological (e.g., task-related, threatening, pleasant) properties. Rauthmann et al. (2015) further state that “situations only have consequences for people's thinking, feeling, desiring, and acting through the psychological processing they receive” (p. 372). Thus, people's individual experiences of psychological situations may deviate from how these situations are experienced by most other people (reality principle). This assumption aligns with the *TESSERA* framework conceived by Wrzus and Roberts (2017). According to the authors, events and single situations can trigger expectancies about how to act and adjust in similar situations. These expectancies then determine which state occurs after the corresponding trigger by choosing a response from a variety of possible states (Wrzus and Roberts, 2017). Conjointly, two people can perceive the same situation or event very differently, leading to diverse reactions and psychological meanings.

A first step toward this important distinction was proposed by Luhmann et al. (2020), who aimed to systematically examined the effects of life events on psychological outcomes. To do so, the authors proposed a dimensional taxonomy which that considers nine perceived characteristics of major life events. I this way, the study uniquely emphasizes the difference between assessing the mere occurrence of a critical life event and taking into account subjective appraisal. However, significantly more research is needed to fully explore how this causes lasting personality trait change.

In conclusion, two aspects of person-situation transactions should be highlighted. First, one situation can be interpreted very differently by two individuals. Expectations and individual goals—as well as variable expressions of personality traits—influence the extent to which a situation is perceived as meaningful and, therefore, determine how people approach it (Bleidorn, 2012; Denissen et al., 2013, 2018). Second, this is also true for life events. Two people can reasonably experience the same major life event as completely differently. Therefore, we focus the present study on the valence of two distinct life events and use this characteristic as our central parameter. In particular, in emerging adulthood, individuals might perceive the behavioral expectations and demands associated with a life event as more pressing than others (Pusch et al., 2018). What remains less clear is how situational perceptions affect personality change after a major life event, but with respect to the current string of literature, it seems reductive to only ask if, but not how, critical life events are experienced.

### The Moderating Role of Mindset

In the previous section, we examined how diverse critical life events can be perceived. Here, we extend our theoretical approach by focusing on the underlying processes that might account for the different perception and spotlight causes of individual personality trait changes. One construct that is highly relevant to the aforementioned regulatory mechanisms is the individual belief system mindset. According to Dweck (1999), an individual's mindset refers to the implicit belief about the malleability of personal attributes. Dweck (1999) distinguishes between growth and fixed mindsets. The growth mindset emphasizes the belief that attributes like intelligence and personality are changeable. Conversely, the fixed mindset refers to the belief that such attributes are immutable. According to Dweck (2012), the individual mindset is not static and can be changed throughout one's life. Actively changing one's mindset toward a growth mindset was found to decrease chronic adolescent aggression, enhance people's willpower, and redirect critical academic outcomes (Dweck, 2012; Yeager et al., 2019). Moreover, Blackwell et al. (2007) found that the belief that intelligence is malleable (incremental theory) predicted an upward trajectory in grades over 2 years of junior high school, while the belief that intelligence is fixed (entity theory) predicted a flat trajectory. Yet, according to a meta-analysis from Sisk et al. (2018), mindset interventions for academic achievement predominately benefitted students with low socioeconomic status or who are at-risk academically. Mindset has also been linked to business-related outcomes (e.g., Kray and Haselhuhn, 2007; Heslin and Vandewalle, 2008). That is, individuals with a growth mindset tend to use “higher-order” cognitive strategies and adapt to stress more easily (Heslin and Vandewalle, 2008). Likewise, mindset has been linked to health outcomes and even mental illness, with the assumption that a growth mindset buffers against psychological distress and depression (e.g., Biddle et al., 2003; Burnette and Finkel, 2012; Schroder et al., 2017). Therefore, a growth mindset can be considered a predictor of psychological resilience (Saeed et al., 2018).

With regard to changes in personality traits, the findings have been mixed. Hudson et al. (2020) investigated college students' beliefs by adapting a personality measure into a mindset measure and administering it within a longitudinal study. They found that the mere belief that personality is malleable (or not) did not affect trait changes. However, in her Unified Theory of Motivation, Personality, and Development, Dweck (2017) suggests that basic needs, mental representations (e.g., beliefs and emotions), and action tendencies (referred to as BEATs) contribute to personality development. Dweck further argues that mental representations shape motivation by informing goal selection and subsequently form personality traits by creating recurring experiences (Dweck, 2017). Thus, there might be more information about indicators such as the integration of mindset, motivation, and environmental influences necessary to understand how personality traits change according to belief systems.

In summary, there is evidence that a belief in the malleability of global attributes allows individuals to adapt to life circumstances in a goal-directed way and that individuals' mindsets determine responses to challenges (Dweck and Leggett, 1988). Building upon the existing literature around environmental influences on personality traits and the diverse effects of mindset, we argue that after experiencing a critical life event, individuals with a growth mindset will adapt to a new situation more easily and accordingly exhibit greater change in relating personality traits. In contrast, individuals with a fixed mindset might react in a more rigid way to unknown circumstances and thus don't experience the need adapt, resulting in no personality trait change.

### The Present Study

This study aims to contribute to the literature around external and internal influences on personality development in emerging adulthood by analyzing changes in the Big Five, the influences of the occurrence of life events *per se* vs. their subjective perception, and the possible moderating effects of mindset in a longitudinal study with a large sample. Most prior studies have focused on personality development in adulthood (e.g., Roberts and Jackson, 2008; Lucas and Donnellan, 2011; Wrzus and Roberts, 2017; Damian et al., 2018; Denissen et al., 2018), but emerging adulthood is marked by tremendous changes; thus, we focus our analyses on this period. According to Arnett (2000, 2007), emerging adulthood is considered a distinct stage between adolescence and full-fledged adulthood. This is seen as a critical life period because it is characterized by more transformation, exploration, and personality formation than any other life stage in adulthood (Arnett, 2000; Ziegler et al., 2015; Bleidorn and Schwaba, 2017). With regard to beliefs systems, Yeager et al. (2019) argue that beliefs that affect how, for example, students make sense of ongoing challenges are most important and salient during high-stakes developmental turning points such as pubertal maturation. For this reason, it is particularly compelling to investigate environmental influences such as major life events that shape the trajectory of personality trait change in emerging adulthood.

To do so, we examined whether two major critical life events (graduating from school and moving away from home) affect personality development. We chose these two major life events because they are uniquely related to emerging adulthood and because existing research has found mixed results regarding their influence on personality trait change (e.g., Lüdtke et al., 2011; Specht et al., 2011; Pusch et al., 2018). Based on prior findings, we constructed three hypotheses. First, we expect that an increase in personality trait change will occur in individuals who graduate from school/move away from home but not in those who did not experience such events. Second, subjective perceptions of the two critical life events will influence personality trait changes in the Big Five. Third, we look at the underlying processes that influence personality and argue, that mindset will moderate the impact of the two stated life events/perception of life events on personality trait change.

## Methods

### Sample and Procedure

For this study, we created the German Personality Panel (GEPP) by collecting data from a large German sample in cooperation with a non-profit online survey provided by *berufsprofiling.de*. This organization assists emerging adults by providing job opportunities and post-graduation academic pathways. After completing the questionnaire, participants received feedback and vocational guidance. In 2016 and 2017, a total of 11,816 individuals between 13 and 30 years old (*M* = 17.72 years; *SD* = 3.22, 50.71% female) took this survey. We used this first round of data-gathering as our longitudinal measurement occasion T1. If participants consented to be contacted again, we reached out via email in October 2018 to request their participation in a second survey. A total of 1,679 individuals between 14 and 26 years old (*M* = 17.39, *SD* = 2.37, 64.82% female) agreed to participate and filled in a second online survey (second measurement occasion of GEPP, T2). The test battery at T2 took approximately 30–40 min, and we provided personalized feedback on personality development, as well as a monetary compensation, to all participants.

Because we were interested in emerging adults who were about to graduate from school?and thus found themselves in a critical time period?we excluded all participants older than 21 at T2. On the other hand, we included 14-year-old participants because they could have entered school in Germany at the age of five and thus graduated from secondary school and/or moved away from home by this age. At T2, 12% had not yet finished school, 32% held a secondary school certificate, and 57% held a university entrance diploma.

To further improve data quality, we obtained an indicator for careless responding by asking about self-reported diligence (“Did you work conscientiously on the test?”). Participants were informed that their answer had no impact on their compensation. At T2, 41 (3%) participants answered “No.” After excluding participants meeting this criterion, a sample of *n* = 1,243, aged 14–21 years (*M* = 16.92, *SD* = 1.75, 67.23% women), remained for subsequent data analyses. All data and further materials are available via osf (https://osf.io/xc6d4/?view_only=5b913c97553d48a290b75a3f725aca3d).

### Sample Attrition

Numerous email accounts were invalid at the second measurement point—for example, because students' personalized school email accounts were deleted following their graduation or because certain institutions used only a single email account to offer vocational counseling to college students (*N* = 3,495). Those who did not participate at the second measurement point (dropouts) were slightly younger than those who participated (continuers) [*M*(ageD) = 17.39; *M*(ageC) = 17.76; *p* ≤ 0.000, *d* = −0.12] and more women filled in the second questionnaire (dropouts = 50.9% women, continuers = 64.8% women; *p* ≤ 0.000, *d* = 0.31). Only modest selectivity effects (measured by Cohen's *d*) in terms of mean differences in personality traits between dropouts and continuers were found at T1; thus, there was negligible systematic attrition (Specht et al., 2011; Pusch et al., 2018). Continuers had slightly higher scores in agreeableness (*d* = 0.17), conscientiousness (*d* = 0.19), and openness (*d* = 0.16) than dropouts, but they almost identical in terms of extraversion (*d* = −0.08) and emotional stability (*d* = 0.01).

### Measures

#### Personality

Personality traits were assessed on both measurement occasions using a short version of the Big Five personality inventory for the vocational context (TAKE5; S&F Personalpsychologie Managementberatung GmbH, 2005). The TAKE5 has been shown to be a highly reliable and valid personality measure (Mussel, 2012). In the short version of the test, each of the Big Five subscales (openness to experience, conscientiousness, extraversion, agreeableness, and emotional stability) consists of three items and was measured on a 7-point Likert scale, ranging from 1 (*strongly disagree*) to 7 (*strongly agree*). Example items for conscientiousness include (translated from German): “Nothing can stop me from completing an important task,” “People around me know me as a perfectionist,” and “My work is always carried out the highest quality standards.” Items were selected to cover the different aspects of each domain therefore internal consistencies provide no valuable indicator. Test-retest reliabilities for the TAKE5 between T1 and T2 were 0.69 for extraversion, 0.52 for openness to experience, 0.57 for conscientiousness, 0.58 for agreeableness, and 0.50 for emotional stability. Small to moderate reliability levels can be explained by the heterogeneity of the items and our attempt to capture rather broad personality constructs. Similar results have been reported for other brief personality scales (Donnellan et al., 2006; Rammstedt et al., 2016). All descriptive statistics and correlations can be found in Table 1, and bivariate correlations of all items can be found at osf (https://osf.io/xc6d4/?view_only=5b913c97553d48a290b75a3f725aca3d).

**Table 1:** Correlations and descriptive statistics among variables.

	**Correlations**															
**Variables**	***N***	***M***	***SD***	**1**	**2**	**3**	**4**	**5**	**6**	**7**	**8**	**9**	**10**	**11**	**12**	**13**
1 Extraversion T1	1,243	4.25	1.22													
2 Agreeableness T1	1,243	4.49	1.24	0.05^*^												
3 Openness T1	1,243	4.19	0.94	0.26^***^	0.11^***^											
4 Emo. Stability T1	1,243	4.18	1.09	0.15^***^	0.39^***^	0.07^*^										
5 Conscientiousness T1	1,243	4.53	1.15	0.10^***^	0.10^***^	0.22^***^	0.19^***^									
6 Extraversion T2	1,243	4.39	1.29	0.68^***^	0.05	0.20^***^	0.12^***^	0.05^*^								
7 Agreeableness T2	1,243	4.50	1.33	−0.03	0.57^***^	0.05^*^	0.23^***^	0.05^*^	0.02							
8 Openness T2	1,243	4.47	0.95	0.23^***^	0.06	0.51^***^	0.08^**^	0.10^***^	0.29^***^	0.09^**^						
9 Emo. Stability T2	1,243	4.25	1.29	0.12^***^	0.28^***^	0.00	0.50^***^	0.10^***^	0.19^***^	0.39^***^	0.10^***^					
10 Conscientiousness T2	1,243	4.78	1.12	0.08	0.06^*^	0.10^***^	0.11^***^	0.57^***^	0.10^***^	0.06^*^	0.14^***^	0.12^***^				
11 Mindset	1,243	3.62	1.45	0.00	0.10^***^	0.15^***^	0.08^**^	0.09^**^	0.04	0.15^***^	0.14^***^	0.13^***^	0.04			
12 Life Event 1	1,030	5.48	1.43	0.05	0.03	0.02	0.12^***^	0.07	0.03^*^	0.06	0.03	0.17^***^	0.04^**^	0.06^*^		
13 Life Event 2	698	4.75	1.51	0.06	0.01	0.07^*^	0.03^*^	0.09^*^	0.03	0.00	0.08^*^	0.09^**^	0.00	0.04	0.17^***^	

*N = Sample size, M = Mean, SD = Standard deviation, Life Event 1 = Perception of graduating from school, Life Event 2 = Perception of moving away from home*,

*** *p < 0.001;*

** *p < 0.01;*

* *p < 0.05*.

#### Life Events

In the present study, we focus on two major life events that are highly characteristic of the critical period between the late teens and young adulthood (Arnett, 2000; Lüdtke et al., 2011; Bleidorn, 2012): moving away from home and graduating from school. At T2, after completing the personality questionnaire, participants rated their subjective perception of each of the two life events on a dimensional 7-point Likert scale (1 = *very negatively*, 7 = *very positively*). Of the initial sample, 68.38% of the participants had graduated from school, 47.66% had moved away from home, and 46.96% had experienced both life events. Participants who had graduated from school were older (*M* = 17.32 years, *SD* = 1.84, female = 68.80%) compared to those who had not yet finished school (*M* = 15.30 years, *SD* = 1.09, female = 68.21%). Those who had moved away from home were approximately 1 year older (*M* = 17.53, *SD* = 1.89, female = 69.30%) compared to those did not yet moved away (*M* = 16.29, *SD* =1.69, female = 66.91%). To avoid potential confounding effects, we only asked about events that had happened within the past year (after the first measurement occasion). This allowed us to account for experiences that took place before T1.

In the second step, in order to obtain a fuller picture, participants also had the option of rating an additional significant life event from a list of 18 potential life events from various domains—such as love and health—based on the Munich Life Event List (MEL; Maier-Diewald et al., 1983). However, the number of individuals who experienced these other life events was too small to allow for further analyses.

#### Mindset

Participants' mindset was measured with a questionnaire based on Dweck's Mindset Instrument (DMI). The 16-item DMI was developed and created by Dweck (1999) and is used examine how students view their own personality and intelligence. In the current study, only items concerning beliefs about the malleability of personality were used. The mindset inventory items were “Personality traits are something a person cannot change,” “You have a certain personality and you really can't do much to change it,” and “You can learn new things, but you can't really change your basic personality.” At T2, participants were presented a 7-point response scale, ranging from 1 (*strongly disagree*) to 7 (*strongly agree*) (*M* = 3.60, *SD* = 1.45). Items were reversed such that higher levels indicated a growth mindset. This short inventory was found to be highly reliable (*M* = 3.60, *SD* = 1.45, ω = 0.81, 95% CI [0.70, 0.84]).

### Statistical Analyses

Analyses were carried out in four steps. First, we conducted confirmatory factor analyses to test for measurement invariance across time points T1 and T2. Second, we constructed latent difference score models for all Big Five scales to test for mean differences in personality traits. Third, we investigated the impact of the life events moving away from home and graduating from school, as well as the perception of these two events on changes in the Big Five. Fourth, we added mindset as a moderator to the model. All statistical analyses were carried out in R and R Studio 1.2.1335 (R Core Team, 2018).

#### Measurement invariance

To ensure that the same construct was being measured across time, we first tested for measurement invariance. For weak measurement invariance, we fixed the factor loadings for each indicator to be equal across measurement occasions and compared this model to the configural model, where no restrictions were applied. The same procedure was followed to assess strong measurement invariance, with the weak invariant model compared to a model with constrained intercepts to equality across time (e.g., the same intercept for Item 2 at T1 and Item 2 at T2) (Newsom, 2015). To evaluate the model fit, comparative fit index (CFI), Tucker–Lewis index (TLI), root mean square error of approximation (RMSEA), and standardized root mean square residual (SRMR) were inspected. Good fit was considered to be indicated when CFI and TLI values were 0.90 or higher, RMSEA below 0.08, and SRMR values below 0.05 (Hu and Bentler, 1999; Marsh et al., 2005). The configural model showed good fit for all of the Big Five traits (All χ^2^[4 24], *df* = 5, *CFI* > [0.98 1.00], *TLI* > [0.94 1.00], *RMSEA* < [0.0 0.06], *SRMR* < [0.0 0.02]). Model fit for partial strong measurement invariance revealed similar fit (all χ^2^[9 50], *df* = 8, *CFI* > [0.96 1.00], *TLI* > [0.92 1.00], *RMSEA* < [0.01 0.07], *SRMR* < [0.01 0.03]) when freely estimating the intercept of the first manifest OCEAN item (Cheung and Rensvold, 2002; Little et al., 2007). All further analyses are based on this model and full results for fit indices are presented in Table S1.

#### Latent Change Score Models

To test for changes in personality over time, we applied latent structural equation modeling analysis with the R package lavaan (version 0.5-23.1097; Rosseel, 2012). Required sample size for the specified latent change score model was estimated by the R-toolbox semTools (MacCallum et al., 2006; Jorgensen et al., 2018) for *RMSEA* = 0.05, *df* = 16, α = 0.05, and a statistical power of 90% to *N* = 672 individuals. Therefore, we consider our sample size to be sufficiently large.

**Figure 1:**
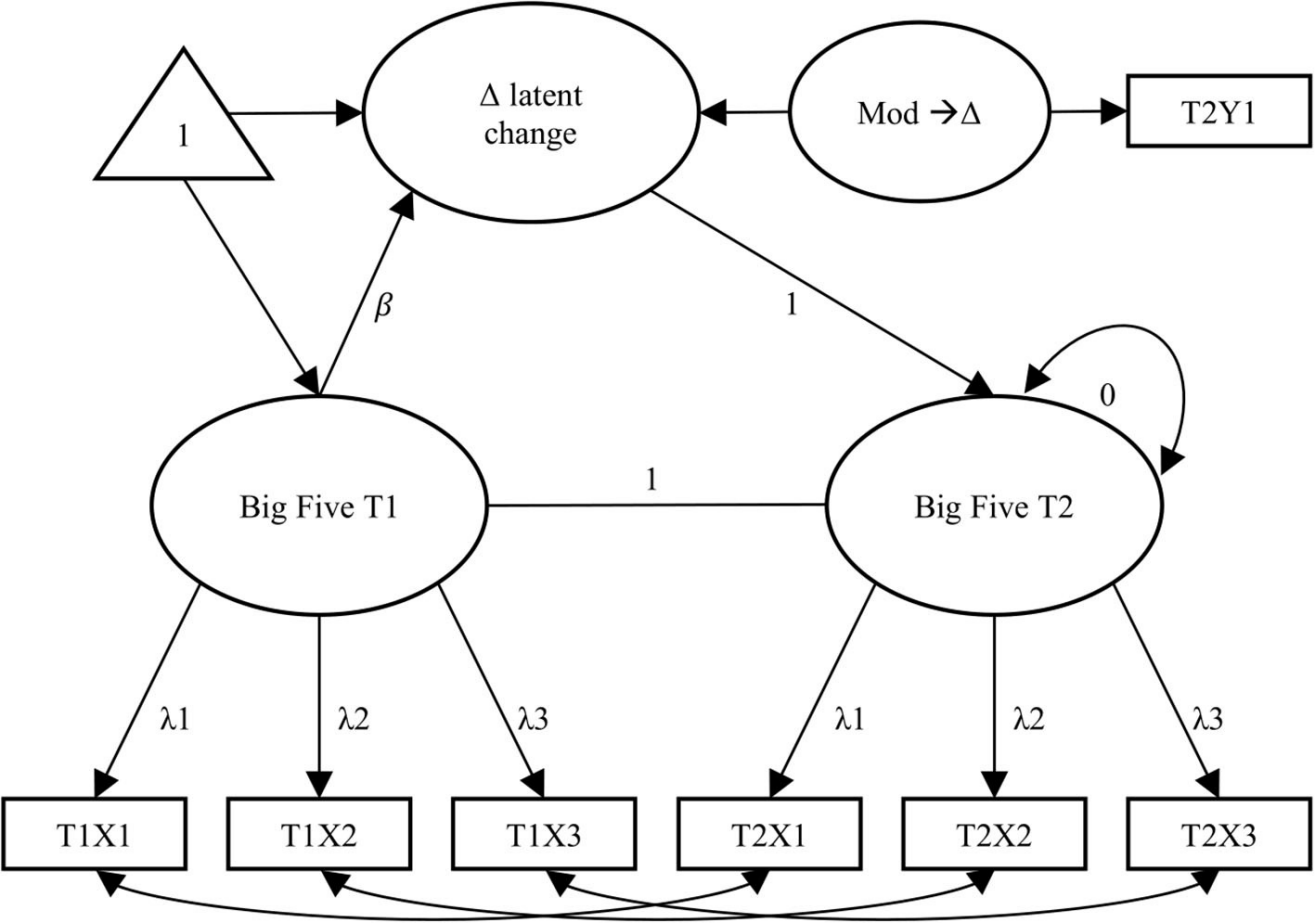
Schematic model of the multiple-indicator univariate latent change score model. The latent construct of interest (each personality trait) was measured at two time points (T1 and T2), using three indicators each time (X1, X2, X3). The lower part of the model constitutes the assessment of measurement invariance. “Δ latent change” captures change from the Big Five trait from T1 to T2. Latent regressions from “Δ latent change” on Mod→ Δ reflect the influence of the covariate *perception of life event* or the moderator *mindset* on the development of the Big Five. Straight arrows depict loadings and regression coefficients, curved arrows co-variances.

As we were first interested in the rate of change, we built a multiple-indicator univariate latent change score model for each of the Big Five domains (Figure 1). Each latent construct of interest (OCEAN) consisted of three observed measures (X1, X2, and X3) at two waves. Equality constraints were imposed on factor loadings and intercepts (Newsom, 2015). Moreover, the autoregressive path was set equal to 1. The means, intercepts, and covariances at the first occasion and for the difference score factor were freely estimated, and all measurement residuals were allowed to correlate among the sets of repeated measurements (McArdle et al., 2002). We accounted for missing data by applying robust maximum likelihood estimation. Finally, after specifying this basic model, the variables of interest—the occurrence of the life event, perception of the life event, and the moderator mindset—were added to the model.

## Results

### Big Five

**Figure 2:**
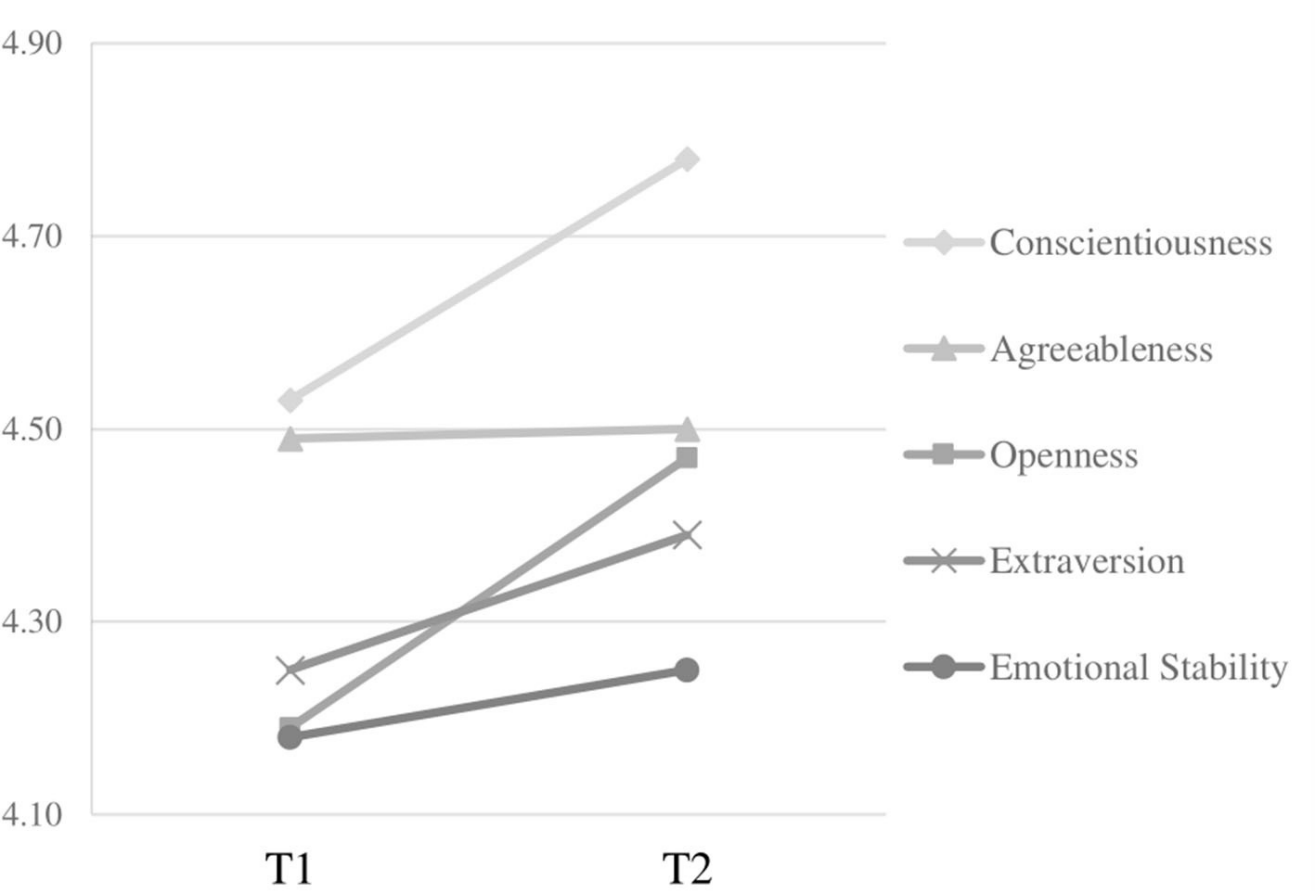
Mean-level changes in Big Five dimensions over measurement occasions T1 and T2.

Standardized mean differences were calculated as an average of all intra-individual increases and decreases in a given personality trait over time. As illustrated in Figure 2, all latent mean scores for the Big Five increased from T1 to T2. Conscientiousness and openness to experience exhibited the largest mean-level changes from T1 to T2, whereas agreeableness (*d* = 0.02) and emotional stability (*d* = 0.07) remained nearly the same. To test for changes in personality, we employed a multiple-indicator univariate latent change score model. Separate models for each of the Big Five all fit the data well (all *CFI* > 0.95, *TLI* > 0.93, *RMSEA* < 0.05, *SRMR* < 0.04). Inspecting the intercepts of the change factors revealed that all Big Five scores changed between T1 and T2, with less increase among individuals with high compared to low levels at T1. The latent means for each personality dimension at each time point, along with their fit indices, are reported in Table 2.

**Table 2:** Big Five mean-level change from T1 to T2 with fit indices, n = 1,243.

**Big Five**	***M* T1**	***M* T2**	***d***	**χ^**2**^(*df*)**	***p*(χ^**2**^)**	***CFI***	***TLI***	***RMSEA***	***RMSEA 90%CI***	***SRMR***	**μΔ**
Extraversion	4.25	4.39	0.11^***^	23.56 (10)	0.00	0.99	0.90	0.03	[0.03–0.06]	0.03	0.73^***^	
Agreeableness	4.49	4.50	0.01	27.28 (10)	0.00	0.99	0.99	0.04	[0.01–0.05]	0.03	1.38^***^	
Openness	4.19	4.47	0.30^***^	52.04 (10)	0.00	0.95	0.93	0.06	[0.04–0.07]	0.04	2.19^***^	
Emotional Stability	4.18	4.25	0.06	45.77 (10)	0.00	0.97	0.96	0.05	[0.02–0.05]	0.04	0.81^***^	
Conscientiousness	4.53	4.78	0.22^***^	10.70 (10)	0.00	1.00	0.99	0.01	[0.00–0.03]	0.02	1.63^***^	

*M T1, Mean at measurement occasion 1; M T2, Mean at measurement occasion 2; d, (mean at Time 2 – mean at Time 1)/baseline standard deviation; χ^2^, chi square difference statistic; df, degrees of freedom; p(chi2), significance of chi square difference statistic; CFI, Comparative Fit Index; TLI, Tucker–Lewis index; RMSEA, root mean square error of approximation; SRMR, standardized root mean square residual; μΔ, intercept of latent change score; p(μΔ), significance of latent change score;*

*** *p < 0.001;*

** *p < 0.01;*

* *p < 0.05*.

### Life Events and Perception of Life Events

To assess personality trait change resulting from experiencing a life event, we included a standardized dichotomized variable “experiencing the life event vs. not” into the model. Again, the model fit the data well for both critical life events (all CFI > 0.94, TLI > 0.92, RMSEA < 0.05, SRMR < 0.04). However, comparing participants who had experienced one of the critical life events (moving away from home or graduating from school) to those who had not revealed that neither life event had a significant impact on changes in personality traits between T1 and T2 (*p* >0.05).

To assess personality trait change resulting from perception of a life event, we included the standardized variable “perception of the life event” for each of the two events into the model and regressed the latent change score on the covariate. This time, results regarding the subjective perception of the life event graduating from school indicated a significant impact on personality change for emotional stability (χ^2^[16] = 94.07, *CFI* = 0.92, *TLI* = 0.90, *RMSEA* = 0.07, *SRMR* = 0.05, λ = 0.05, *p*[λ] < 0.05). Specifically, participants who had experienced graduating from school more negatively exhibited a diminished increase in emotional stability than compared to individuals who had experienced graduating from school more positively. We also found evidence that subjective perceptions are relevant for extraversion. A greater positive change in extraversion was observed when participants experienced graduating from school more positively than compared to negatively (χ^2^ [16] = 23.90, *CFI* = 0.99, *TLI* = 0.99, *RMSEA* = 0.02, *SRMR* = 0.03, λ = 0.10, *p*[λ] = 0.05). Subjective perceptions moving away from home had no impact on trait changes in any of the Big Five traits. Descriptive statistics for the life events along with model fit indices can be found in Table S2.

### Mindset

**Figure 3:**
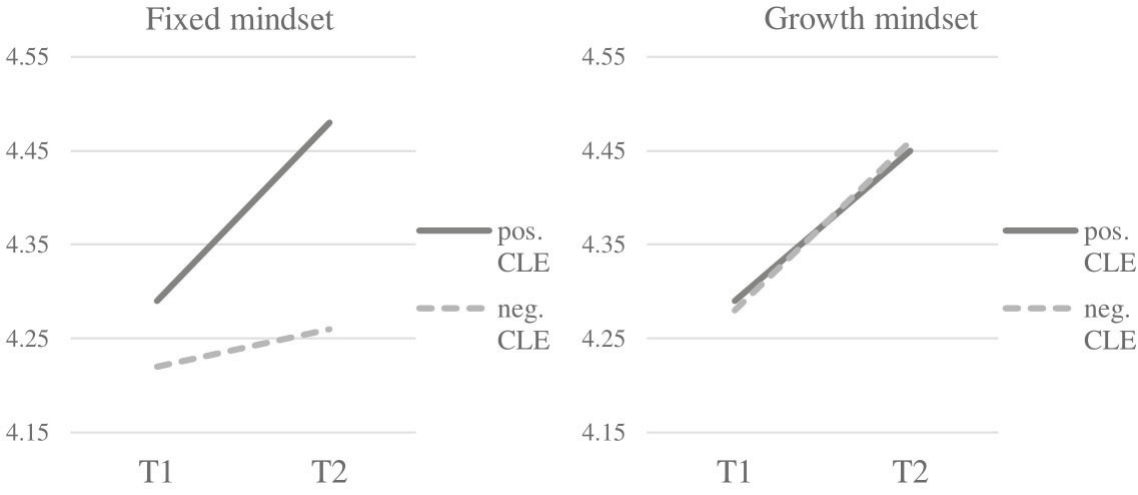
Change in trait extraversion for people with a fixed vs. growth mindset with regard to the perception of life event *graduation from school*.

To test for a moderating role of mindset, an interaction term between mindset and each of the two critical life events was constructed. First, we built an interaction term between mindset and the dichotomous variable “experienced the life event” and regressed the latent change factor on the interaction term. Separate models for each of the Big Five all fit the data well (all CFI > 0.94, TLI > 0.92, RMSEA < 0.05, SRMR < 0.05). As shown in Table S3, no effects for the Big Five traits were significant for the distinction between experienced the life event vs. did not experience the life event (*p* > 0.05). Second, for each of the two life events an interaction term between mindset and perception of the life event was built analogously. For extraversion, we found a significant influence of the moderator when assessing the perception of graduating from school (χ^2^ [16] = 25.62, *CFI* = 0.99, *TLI* = 0.99, *RMSEA* = 0.03, *SRMR* = 0.03, λ = −0.09, *p*[λ] = 0.05). Hence, a fixed mindset indicates less change in extraversion when experiencing the critical life event graduation from school. More specifically, regarding manifest means of extraversion, participants with a growth mindset experienced almost the same amount of increase in extraversion over time, regardless of their perception (positive or negative) of the critical life event. On the other hand, participants with a fixed mindset only show an increase in extraversion when they experienced the life event more positively (see Figure 3). No effects for the interaction between mindset and the critical life event moving away from home were significant.

## Discussion

The purpose of the present study was to investigate the effect of external sources such as life events and internal dispositions like the mindset on personality trait change. We assert that exploring whether the subjective experience of life events is associated with personality trait development constitutes an important future directions in various domains of personality research. Therefore, we took a closer look at the underlying processes, particularly as they relate to individual differences in situational perceptions and belief systems. We investigated how two critical life events (moving away from home and graduating from school) influence personality trait change, the role of subjective perceptions of these events, and how internal belief systems like mindset moderate the impact of life events on trait change.

### Mean-Level Change

Since our sample was selected to be between 14 and 21 years of age, most of our participants were classified as emerging adults Arnett, 2000, 2007. A large body of research has consistently demonstrated that emerging adulthood is characterized by trait changes related to maturity processes (for an overview, see Roberts et al., 2006). Thus, emerging adults tend to experience increases in conscientiousness, emotional stability, openness, and (to a lesser degree) agreeableness. This pattern is often called the “maturity principle” of personality development, and it has been found to hold true cross-culturally (Roberts and Jackson, 2008; Bleidorn, 2015). Although the effects were small, we found evidence for mean-level changes in line with the maturity principle and functional personality trait development. Extraversion, openness, agreeableness, conscientiousness, and emotional stability significantly increased over the 1-year period. The largest changes were found for openness and conscientiousness. These changes are most likely to be explained by attempts to satisfy mature expectations and engage in role-congruent behavior. While increases in openness might be due to identity exploration, higher scores on conscientiousness could reflect investment in age-related roles. Individuals might, for instance, take increased responsibility for social or career-related tasks that require more mature functioning (Arnett, 2000, 2007).

### Life Events

First, we analyzed whether the occurrence of a life event *per se* had an influence on personality trait change. In our study, neither of the critical life events?moving away from home or graduating from school?affected Big Five trait change over the two measurement occasions. One possible explanation is that the two chosen life events were not prominent enough to evoke far-reaching changes in personality traits (Magnus et al., 1993; Löckenhoff et al., 2009). In line with a study by Löckenhoff et al. (2009), more stressful, adverse events might have triggered more pronounced and predictable effects on personality traits. Moreover, the period between the late teens and early adulthood is characterized by a large number of stressful events and daily hassles (Arnett, 2000, 2007). In a comprehensive review of emerging adulthood by Bleidorn and Schwaba (2017), graduates also experienced changes in other personality traits, such as openness and emotional stability, which suggests that many developmental tasks and major life transitions contribute to changes in Big Five trait domains. Furthermore, according to Luhmann et al. (2014) and Yeager et al. (2019), life events may not only independently influence the development of personality characteristics, they might also interact with one another. Researchers must address the interpretation of other challenges that adolescents experience. This notion is also supported in a study by Wagner et al. (2020), who introduced a model that integrates factors that are both personal (e.g., genetic expressions) and environmental (e.g., culture and society). The authors assert that the interactions and transactions of multiple sources are responsible for shaping individuals' personalities, and, in order to understand how they interact and develop over time, more integrated research is needed. Future studies should focus on a wider range of important life events and environmental influences during emerging adulthood and account for possible accumulating effects.

Second, and perhaps most remarkably, our findings revealed a different picture after we analyzed how the two critical life events were perceived. When participants experienced graduating from school negatively, a greater decrease in emotional stability was observed. Conversely, when the event was evaluated positively, a greater positive change in extraversion was reported. There are clear theoretical links between these two traits and the perception of life events in terms of emotional valence. While low emotional stability encompasses a disposition to experience negative emotions such as fear, shame, embarrassment, or sadness (especially in stressful situations), extraverted individuals are characterized by attributes such as cheerfulness, happiness, and serenity (Goldberg, 1990; Depue and Collins, 1999). In line with the notion of a bottom-up process of personality development (Roberts et al., 2005), experiencing a major life event as either positive or negative might lead to a prolonged experience of these emotions and, thus, ultimately to altered levels of the corresponding personality traits. These findings are in line with previous research on subjective well-being (SWB). In fact, variance in SWB can be explained by emotional stability and extraversion, indicating a robust negative relationship between low emotional stability and SWB and a positive relationship between extraversion and SWB (Costa and McCrae, 1980; Headey and Wearing, 1989). Moreover, Magnus et al. (1993) found selection effects for these traits, suggesting that high scorers in extraversion experience more subjectively positive events, and low scorers in emotional stability experience many (subjectively) negative events (see also Headey and Wearing, 1989).

### Mindset

In the present study, we found evidence of a moderating influence of mindset on the impact of the life event graduating from school for the trait extraversion. Our results indicate that people with a growth mindset show greater change in extraversion, almost regardless of whether they experienced the life event more negatively or more positively. On the other hand, the present results indicate that people with a fixed mindset show an increase in extraversion after experiencing a life event more positively, but almost no change in extraversion when experiencing graduating from school negatively.

Interestingly, we only found effects for extraversion. As previously mentioned, trait extraversion stands for behavioral attributes such as how outgoing and social a person is, and this is related to differences in perceived positive affect (Goldberg, 1990; Magnus et al., 1993; Roberts et al., 2005). The characteristics of extraversion can be linked to the assumption that people with a growth mindset show greater resilience (Schroder et al., 2017; Yeager et al., 2019), especially in the face of academic and social challenges (Yeager and Dweck, 2012). Thus, people who believe that their internal attributes are malleable confront challenges such as graduation by adapting and learning from them; our findings suggest that this results in an increase in extraversion. By contrast, people who believe that they cannot change their personality characteristics might attribute a negatively experienced graduation to external circumstances out of their control. Thus, they do not rise from a negative life event and experience no impetus to become more extraverted.

The above notwithstanding, more research is needed, as we found no evidence for the other Big Five personality traits. Further, the relationship between mindset and personality is complex to disentangle. We examined only two major life events in this first attempt. More attention is needed with respect to other life events and their interplay with internal belief systems and implicit theories to explore possible far-reaching effects on behavior.

In summary, the present study makes an important contribution to the literature on personality development in emerging adulthood with a special focus on external and internal influences and the assessment of critical life events. Our findings support the notion of a dimensional approach to life events, as introduced by Luhmann et al. (2020), in contrast to a typological approach. With regard to research on situational perception, it seems reductive to examine the occurrence of certain life events rather than their subjective perceptions. As previously mentioned, many studies emphasize that (1) events and single situations can trigger expectancies about how to act and adjust in similar situations (TESSERA framework, Wrzus and Roberts, 2017); (2) psychological situations and person-situation transactions deviate from one another (Rauthmann et al., 2015); and (3) regulatory mechanisms influence the variability in individual personality trait change (Denissen et al., 2013).

Again, further research is needed to explore the underlying processes behind critical life events and their impact on personality trait changes. In doing so, great care should be taken in selecting life events with a strong social and emotional component with respect to individual perceptions. Finally, there is also a need for more research into the selection of life events being assessed with regard to their interplay.

## Limitations and Future Directions

Our research demonstrates the importance of examining the underlying processes behind personality changes that arise from external influences such as life events. One of the strengths of this study was our large sample, which comprised *N* = 1,679 German emerging adults and allowed us to use powerful statistical methods. One limitation was that we gathered data across a 1-year time interval with only two measurement occasions. As noted by Luhmann et al. (2014), the inclusion of more than two measurement points makes it easier to distinguish between sudden short- or long-term shifts and more gradual linear changes. With this in mind, it is possible that critical life events correlate with temporary disruptions of personality maturation; tracing the impact of a single life event on personality trait change might not be as straightforward as is often assumed. Moreover, two measurement occasions can only reveal the immediate effect of life events on personality traits and may, therefore, neglect long-term effects that become salient after more time has passed. Future studies should also incorporate more characteristics of life events. We concentrated our study on the valence of critical life events, but other features—such as impact, challenge, and predictability—could reveal a more comprehensive picture (Luhmann et al., 2020).

Another limitation of the present study is that all our data relied on self-report personality measures. Even though almost all research on personality change is based on self-report measures, the influence of (for example) self-concepts cannot be neglected. Self-reported data might thus depart from other types of data in terms of differential stability, for example (Wagner et al., 2020). Hence, changes in the Big Five domains might reflect subjective rather than observable changes in personality. At the same time, we believe that our approach of assessing personality traits and the perception of life events gives valuable insights into personality development, since we focused on how individuals consciously understand their experiences. Nevertheless, it would be informative to compare both approaches (observer and self-reported data) to examine how they complement one another (see also: Bleidorn et al., 2020).

Yet another important issue that must be mentioned are our attrition effects. As previously stated, the data for the first measurement occasion was gathered through a non-profit self-assessment test intended to help students explore post-graduation occupational opportunities. Hence, our sample might be prone to selection effects and confounding preexisting differences: only emerging adults who were concerned about their future might have taken the test in the first place. The self-selection to voluntarily participate in a research study might also explain the higher percentage of female participants. Moreover, some of the Big Five traits from T2 dropouts were correlated with T1 personality traits. Therefore, our results should be interpreted with caution; participants with low conscientiousness, for example, might have been more likely to drop out or have been excluded from our study due to the diligence check, and thus conscientiousness could have risen over the study period because the sample composition shifted between T1 and T2. Nevertheless, the noted differential attrition effects were rather small and reflect only modest selectivity (see also Lüdtke et al., 2011; Specht et al., 2011).

Finally, we did not examine cultural differences. With our German sample, we only investigated patterns in a modern Western industrialized country. Hence, we did not control for different cultural and demographic backgrounds, and our results might thus not be applicable to a broader range of individuals.

## Conclusion

The present research improves our understanding of personality trait development during the critical period of emerging adulthood and demonstrates the importance of examining the underlying processes behind personality changes that arise from external influences such as life events. We showed how two critical life events can shape and adjust life trajectories, which is a necessary step toward gaining a comprehensive picture of the underlying processes of personality trait change across the life course. In addition to changes in the operationalization of life event research, larger and more diverse samples over more measurement occasions are needed to further explore how individual perceptions and internal belief systems influence our personality during and after experiencing critical life events.

## Data Availability Statement

The datasets generated for this study can be found in online repositories. The names of the repository/repositories and accession number(s) can be found below: All data, further materials, and items are available via OSF at: https://osf.io/xc6d4/.

## Ethics Statement

The studies involving human participants were reviewed and approved by the ethic commission of Julius-Maximilians-Universität Würzburg. The patients/participants provided their written informed consent to participate in this study.

## Author Contributions

JHDV and PM designed the study and formulated the hypotheses. MS and AF provided the testing platform and set up the test battery. JHDV, MS, and AF were responsible for recruiting the sample and administrating the panel. JHDV and PM conducted the data analysis. JHDV designed the figures and drafted the manuscript. All authors discussed the results and commented on the manuscript.

## Conflict of Interest

The authors declare that the research was conducted in the absence of any commercial or financial relationships that could be construed as a potential conflict of interest.
