# Supplementary material for: Personality Development in Emerging Adulthood—How the Perception of Life Events and Mindset Affect Personality Trait Change
Source: Front Psychol. 2021 Jun 10;12:671421. doi: 10.3389/fpsyg.2021.671421 (PMC8256263; doi:10.3389/fpsyg.2021.671421)
Supplement: Supplementary file 1 [file Table_1.pdf]

Supplementary Material

Supplementary Material for the Manuscript: Personality Development in Emerging Adulthood – How the Perception of Life Events and Mindset Affect Personality Trait Change

This file includes:

Table S1, S2, and S3

Table S1.

Fit indices for measurement models with increasing degree of invariance across time, n = 1243

Model	χ2	*df*	*p*(χ2)	CFI	TLI	RMSEA	RMSEA 90% CI	SRMR
Extraversion								
Model 1: Configural invariance	4.47	5	.48	1.00	1.00	.00	[.00 – .04]	.01
Model 2: Metric invariance	9.82	7	.20	1.00	1.00	.02	[.00 – .04]	.02
Model 3: Strong invariance	13.08	8	.00	1.00	1.00	.02	[.00 – .04]	.02
Emotional Stability								
Model 1: Configural invariance	3.95	5	.56	1.00	1.00	.00	[.00 – .03]	.01
Model 2: Metric invariance	20.80	7	.00	.99	.98	.04	[.02 – .06]	.03
Model 3: Strong invariance	21.01	8	.01	.99	.98	.04	[.02 – .06]	.03
Agreeableness								
Model 1: Configural invariance	16.43	5	.01	.99	.98	.05	[.02 – .07]	.02
Model 2: Metric invariance	19.70	7	.01	.99	.99	.04	[.02 – .06]	.02
Model 3: Strong invariance	23.19	8	.00	.99	.98	.04	[.02 – .06]	.02
Conscientiousness								
Model 1: Configural invariance	1.26	5	.94	1.00	1.00	.00	[.00 – .01]	.01
Model 2: Metric invariance	7.41	7	.39	1.00	1.00	.01	[.00 – .04]	.02
Model 3: Strong invariance	9.38	8	.31	1.00	1.00	.01	[.00 – .04]	.02
Openness								
Model 1: Configural invariance	24.92	5	.00	.98	.94	.06	[.04 – .06]	.02
Model 2: Metric invariance	40.32	7	.00	.96	.92	.06	[.04 – .06]	.03

Model 3: Strong invariance	49.77	8	.00	.96	.92	.07	[.05 – .08]	.03

Note. Model fit parameters for testing measurement invariance. The configural model requires all items to load on the same factor; for the weak measurement invariance model the regression weights were fixed across time; for the strong measurement invariance model additionally the intercepts were fixed across time; $\chi 2$ = chi square difference statistic; df = degrees of freedom; $p(\chi 2)$ = significance of chi square difference statistic; CFI = Comparative Fit Index, should be above 0.90; TLI = Tucker–Lewis index, should be above 0.90; RMSEA = root mean square error of approximation, should be below 0.08; RMSEA 90 %CI = 90 % confidence interval of RMSEA; SRMR=standardized root mean square residual, should be below 0.05

Table S2.

Fit indices for measurement models of life events and the perception of life events, n= 1243

Model	$\chi2(df)$	$p(\chi2)$	CFI	TLI	RMSEA	RMSEA 90% CI	SRMR	*CLE →Δ*	*CLE →Δ(p)*	*μΔ*	*μΔ (p)*
Graduating from school yes/no											
Emotional Stability	89.55(16)	.00	.94	.93	.06	[.04 – .07]	.04	-.00	.73	1.33	.00
Extraversion	19.70(16)	.23	1.00	1.00	.01	[.00 – .03]	.02	-.03	.55	.75	.00
Openness	50.84(16)	.00	.96	.95	.04	[.03 – .05]	.03	.01	.85	1.96	.00
Agreeableness	45.62(16)	.00	.99	.98	.04	[.03 – .06]	.03	-.02	.16	1.35	.00
Conscientiousness	13.27(16)	.65	1.00	1.00	.00	[.00 – .02]	.02	-.01	.79	1.51	.00
Moving away yes/no											
Emotional Stability	69.11(16)	.00	.96	.94	.05	[.04 – .06]	.05	-.02	.08	.94	.00
Extraversion	29.36(16)	.00	.99	.99	.03	[.01 – .04]	.03	.05	.25	.77	.00
Openness	58.25(16)	.00	.95	.94	.05	[.03 – .06]	.04	.01	.91	1.96	.00
Agreeableness	41.30(16)	.00	.99	.98	.04	[.02 – .05]	.03	-.02	.23	1.64	.00
Conscientiousness	17.65(16)	.35	1.00	1.00	.01	[.00 – .03]	.02	-.04	.07	1.51	.00
Perception of graduating school											
Emotional Stability	94.07(16)	.00	.92	.90	.07	[.06 – .08]	.05	.05	.02	-.06	.00

Extraversion	23.90(16)	.00	1.00	.99	.02	[.00 – .04]	.03	.10	.05	.76	.00
Openness	43.22(16)	.00	.96	.95	.04	[.03 – .06]	.03	-.01	.90	1.87	.00
Agreeableness	38.49(16)	.00	.99	.98	.04	[.02 – .05]	.03	.03	.23	1.58	.00
Conscientiousness	30.46(16)	.02	.99	.99	.03	[.01 – .05]	.03	.02	.48	1.58	.00
Perception of moving away											
Emotional Stability	56.75(16)	.00	.94	.92	.06	[.05 – .08]	.05	.02	.37	-.07	.01
Extraversion	14.12(16)	.59	1.00	1.00	.00	[.00 – .03]	.02	-.01	.83	.72	.00
Openness	46.41(16)	.00	.94	.92	.05	[.04 – .07]	.04	.02	.74	2.03	.00
Agreeableness	32.46(16)	.01	.98	.98	.04	[.02 – .06]	.03	.01	.81	1.63	.00
Conscientiousness	24.98(16)	.07	.99	.99	.03	[.00 – .05]	.04	-.05	.20	1.70	.00

Note. Model fit parameters and estimates for the latent change model (see Figure 1). $\chi2$ = chi square difference statistic; df = degrees of freedom; $p(\chi2)$ = significance of chi square difference statistic; CFI = Comparative Fit Index, should be above 0.90; TLI = Tucker–Lewis index, should be above 0.90; RMSEA = root mean square error of approximation, should be below 0.08; RMSEA 90 %CI = 90 % confidence interval of RMSEA; SRMR=standardized root mean square residual, should be below 0.05; CLE$\rightarrow\Delta$ = Regression weight lambda of the critical life event, as a measure of how the critical life event/ perception of life event influences changes in the Big Five traits from T1 to T2; CLE$\rightarrow\Delta$ *(p)*= significance of critical life event/ perception of life event; $\mu\Delta$ = intercept of latent change score; $p(\mu\Delta)$ = significance of latent change score

Table S3.

Fit indices for measurement models for life events and mindset, n = 1243

Model	$\chi2(df)$	$p(\chi2)$	CFI	TLI	RMSEA	RMSEA 90% CI	SRMR	*Mod→Δ*	*Mod→ Δ(p)*	*μΔ*	*μΔ (p)*
Graduating from school yes/no											
Emotional Stability	58.49(16)	.00	.97	.96	.05	[.03 – .06]	.04	.01	.54	1.33	.00
Extraversion	24.30(16)	.08	1.00	1.00	.02	[.01 – .04]	.02	.02	.68	.75	.00
Openness	53.66(16)	.00	.96	.94	.05	[.03 – .06]	.04	-.02	.67	1.96	.00
Agreeableness	37.95(16)	.00	.99	.99	.03	[.01 – .04]	.03	-.02	.30	1.65	.00
Conscientiousness	15.80(16)	.47	1.00	1.00	.00	[.00 – .03]	.02	-.02	.44	1.52	.00
Moving away yes/no											
Emotional Stability	66.04(16)	.00	.96	.95	.05	[.04 – .06]	.05	.01	.48	1.33	.00
Extraversion	28.78(16)	.03	.99	.99	.03	[.01 – .04]	.02	-.05	.30	.74	.00
Openness	50.73(16)	.00	.96	.95	.04	[.03 – .06]	.04	-.01	.76	1.96	.00
Agreeableness	31.21(16)	.01	.99	.99	.03	[.02 – .04]	.03	-.01	.75	1.65	.00
Conscientiousness	20.84(16)	.19	1.00	1.00	.02	[.00 – .03]	.03	-.02	.43	1.51	.00
Perception of graduating school											
Emotional Stability	58.00(16)	.00	.96	.94	.05	[.04 –.07]	.04	-.00	.97	1.51	.00

Extraversion	25.62(16)	.06	.99	.99	.03	[.00 – .04]	.03	-.09	.05	.75	.00
Openness	41.62(16)	.00	.96	.95	.04	[.03 – .06]	.04	-.04	.45	1.87	.00
Agreeableness	34.66(16)	.00	.99	.98	.03	[.02 – .05]	.03	.01	.82	1.56	.00
Conscientiousness	20.46(16)	.20	1.00	1.00	.02	[.00 – .04]	.02	-.01	.75	1.57	.00
Perception of moving away											
Emotional Stability	43.03(16)	.00	.96	.95	.05	[.03 – .06]	.05	.01	.42	1.69	.00
Extraversion	24.49(16)	.08	.99	.99	.03	[.00 – .05]	.04	-.09	.15	.70	.00
Openness	45.87(16)	.00	.94	.92	.05	[.04 – .07]	.04	.08	.29	2.12	.00
Agreeableness	19.60(16)	.24	1.00	1.00	.02	[.00 – .04]	.03	.00	1.00	1.63	.00
Conscientiousness	21.24(16)	.17	.99	.99	.02	[.00 – .05]	.03	.03	.31	1.72	.00

Note. Model fit parameters and estimates for the latent change model (see Figure 1). $\chi 2$ = chi square difference statistic; df = degrees of freedom; $p(\chi 2)$ = significance of chi square difference statistic; CFI = Comparative Fit Index, should be above 0.90; TLI = Tucker–Lewis index, should be above 0.90; RMSEA = root mean square error of approximation, should be below 0.08; RMSEA 90 %CI = 90 % confidence interval of RMSEA; SRMR=standardized root mean square residual, should be below 0.05; Mod→Δ = Regression weight lambda of the moderator Mindset, as a measure of how the critical life event influences change in each of the Big Five traits from T1 to T2; Mod→Δ *(p)*= significance of the moderator; $\mu\Delta$ = intercept of latent change score; $p(\mu\Delta)$ = significance of latent change score
